# Laser-assisted tooth extraction in patients with impaired hemostasis

**DOI:** 10.37796/2211-8039.1072

**Published:** 2021-06-01

**Authors:** Elena Vladimirovna Larionova, Ekaterina Yurievna Diachkova, Elena Anatolievna Morozova, Albert Artemovich Davtyan, Svetlana Viktorovna Tarasenko

**Affiliations:** Department of Oral Surgery of E.V. Borovskiy Institute of Dentistry of I.M. Sechenov First Moscow State Medical University (Sechenov University), Russia

**Keywords:** bleeding, erbium laser, hemostasis, platelets, tooth extraction, thrombocytopenia

## Abstract

**Introduction:**

The provision of efficient dental care to patients with hemostatic disorders is tied to difficulties and problems, such as prolonged bleeding after or during surgical manipulation.

**Aim:**

was to increase the efficiency of oral surgery in patients with thrombocytopenia with the use of erbium laser on different stages of tooth extraction.

**Methods:**

Patients (n = 96) were selected for tooth extraction on an outpatient basis: patients with confirmed thrombocytopenia (age 44 ± 15.4, 19–74) were included in the 1^st^ group, and patients without impaired hemostasis (age 47.6 ± 15.3, 19–81) were included in the 2^nd^ group (p > 0.05). In the 1st group, operation with the use of erbium laser with with a wavelength of 2490 nm in a noncontact mode was performed in two stages: separation of a circular tooth ligament and curettage of the socket after tooth extraction. In the 2^nd^ group (control), the treatment was provided according to a traditional algorithm. Before the operation, the lab blood tests for thrombocytes were performed in both groups; moreover, for the 1st group, the duration of bleeding and total coagulation were evaluated. In the postoperative period, pain and edema of soft tissues and hemostasis duration were assessed in both groups.

**Results:**

According to the results of our research, the use of erbium laser in the 1^st^ group allowed us to decrease hemostasis duration compared with the control group of patients (80.9 ± 35.9 and 175 ± 67.5 sec, p < 0.01) and reach the similar probability of postoperative bleeding after tooth extraction (p < 0.5). Pain and edema of soft tissues in dynamics after operation were less in the 1^st^ group (p < 0.001).

**Conclusion:**

Application of erbium laser is an up-to-date method that can be successfully used in surgical treatment in patients with hemostasis failure for bleeding and other postoperative complications prevention and stimulation of the alveolar epithelialization after tooth extraction.

## 1. Introduction

Currently, there are difficulties in providing dental care to patients with diseases of the hematopoietic system, including a lack of preventive orientation and planning in treatment, inadequacy of its quality to modern requirements, and low level of dental hygiene education and patient awareness. All these problems dictate the need to address the issues of providing dental care to patients with this pathology [1–6].

When performing surgical interventions in patients with impaired platelet hemostasis, in conditions such as thrombocytopenia, thrombocythemia, and thrombocytopathy, there is a risk of intra- and postoperative bleeding and hematoma formation, due to a significant decrease or possible increase in platelet count, or a pathological change in platelet function [7–9]. First-line drug treatment in patients with the autoimmune impaired hemostasis alike primary thrombocytopenia includes the appointment of glucocorticoids or cytostatic medicine, which can lead to the development of immunosuppression and influence the repair and regeneration processes, contributing to the occurrence of postoperative inflammatory complications [10–16]. In most cases, dental interventions are usually carried out after preliminary medical preparation, “under the cover” of such hemostatic drugs as, for example, cryoprecipitate, freshly frozen blood plasma and others [17–22]. The development of effective substitution therapy and nonspecific drugs can successfully prevent and treat almost all types of bleeding, and thereby achieve a qualitatively new standard of living for hematological patients. However, in urgent cases, it is necessary to provide surgical dental care without prolonged medical preparation [23–26].

Laser technologies are now widely used methods of treatment of diseases and in medicine, also in dentistry. Dentists can use different lasers according to their modes and wavelengths for different purposes: Remark: Please replace with “ablation of hard tissues of tooth; surgery of oral mucosa, particularly in chronic conditions like ulcers and tissues of periodontum; endodontic dentistry; and dental implantation.” [27, 28].

Earlier, dentists used only the physiotherapeutic effect of the low-intensity lasers, but with the development of medicine, the opportunities of laser application are also widening. The diode laser is characterized by the rather high profile of safety without the damage of the adjunct area besides target points that allow us to use it even during tender manipulations in dentistry as endodontics and periodontics treatment, but it does not show sufficient antibacterial effect in all cases. In oral surgery during the last years the use of high intensity lasers, such as erbium, is considered as an alternative to cutting and rotational instruments but can lead to some unpleasant outcomes, such as tissue overheating. Characteristics of appropriate situations for the use of laser during surgery include the maintenance of a good sterile zone, avoidance of active bleeding, avoidance of disturbance of entire neighborhood structures in the oral cavity. The decrease in the loss of marginal periodontium and in the risk of scar formation after using lasers was noted, particularly in comparison with the traditional scalpel techniques [29]. Also, the erbium laser can create conditions for speeding wound epithelization, stimulation of local immune system elements and reduction of pathogenic microorganism activity [30].

Laser technology used in dentistry allows decreasing the risk of possible complications, duration of manipulation, the level of patient fear on dental visit as well as achieving good postoperative results, which is particularly important for patients with impaired hemostasis because they are scared of possible extensive bleeding and need for hospitalization.

The aim of the study was to increase the efficiency of surgical dental treatment of patients with platelet hemostasis disorders using laser technologies.

## 2. Methods

As part of this study, an examination and surgical treatment of 96 patients requiring surgical dental care were performed during 6 years since 2014 at the Department of Dental Surgery of the Institute of Dentistry of Sechenov University, Moscow, Russia and Department ofMaxillofacial Surgery of Moscow State Medical Dentistry University.

This study was approved by Local Committee of Ethics N⍛6 on 02/22/2011, N⍛ 14–19 on 11/13/2019 and performed according to the principles outlined in the Declaration of Helsinki. We received the patients ‘ consent for using the results of research, X-ray, and photos of the operation.

Two groups of patients were formed: in the 1^st^ group I (n = 48) patients had impaired platelet hemostasis. Surgical dental care for these patients was provided using an erbium laser with a wavelength of 2940 nm. The 2^nd^ group (control group, n = 48) consisted of patients without hemostatic disorders. Surgical dental treatment was performed using traditional methods.

Inclusion and exclusion criteria for participant enrolment in our research are listed in Table 1.

All patients underwent standard dental and radiological examinations. When examining the oral cavity, attention was drawn to the presence of foci of chronic infection (carious lesions, complications of caries), the condition of periodontal tissues, the presence of hemorrhagic syndrome manifestations in the oral cavity (bleeding of the mucous membrane, petechiae, and ecchymosis), and oral hygiene index.

As a laboratory preoperative examination in patients with hemostatic pathology, the following methods were performed: a common blood test (hematology), coagulogram and express diagnostic methods for determination of bleeding time according to Duke (a method that involves a stab incision in patient’s cleaned finger or earlobe with a lancet and later, using a timer, the blood is blotted twice a minute; time is measured in minutes) [31] and time of total blood coagulation according to Morawitz (a drop of blood taken from a finger or earlobe is applied to a glass slide; turning on the stopwatch, a thin glass capillary is lowered into a drop of blood every 20–30 seconds; coagulation time is determined at the time the first thin filament of fibrin appears when the capillary is pulled from a drop of blood) [32].

Patients with platelet hemostasis disorders underwent surgical treatment under local anesthesia on an outpatient basis and according to indications: tooth extraction (due to the chronic apical periodontitis or its exacerbation and acute or chronic pericoronitis with the tooth dystopia, followed by treatment of the extracted tooth sockets with laser radiation in a non-contact mode).

**Fig. 1:**
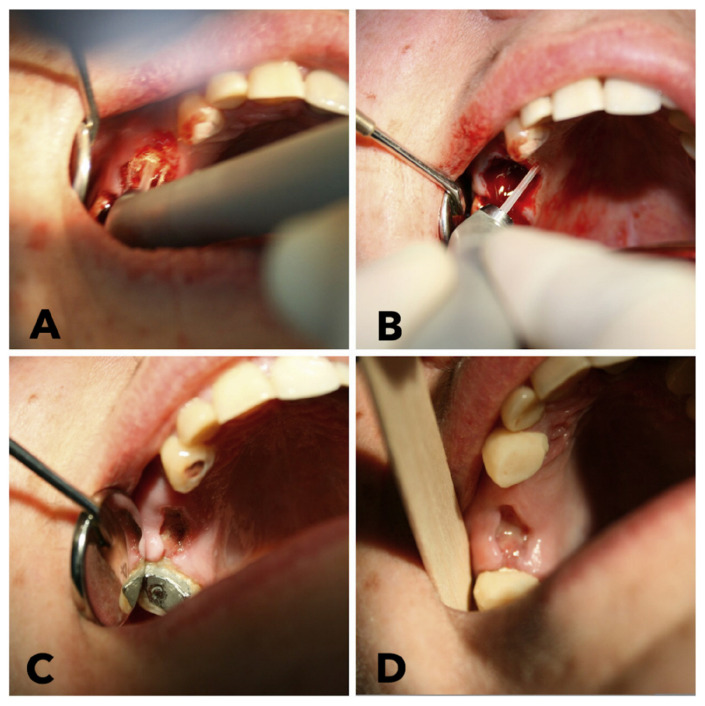
Tooth extraction with erbium laser and postoperative view. (A) Separation of a circular tooth ligament using radiation of the erbium laser with the energy of 300 mJ and a frequency of 10 Hz without water-air cooling around the root of tooth 1.6. (B) Curettage of the socket of the extracted tooth 1.6 root using an erbium laser with the energy 250 mJ and frequency 15 Hz with water-air cooling. (C)The socket of the extracted tooth 1.6 root. (D) The socket of the extracted tooth 1.6 root on the 3^rd^ day after surgery.

In patients with hemostatic disorders, we used an erbium laser with a wavelength of 2940 nm (Fig. 1A), which can be used when working with both soft and hard tissues. Among all the operations we performed, the most frequent was the tooth extraction.

When working with soft tissues, we used radiation energy of 300 mJ and a frequency of 10 Hz without water-air cooling to coagulate the walls of the vessels of soft tissues and ensure hemostasis. Work on hard tissues was performed by an erbium laser in a noncontact manner in a pulsed mode, the radiation energy was 250 mJ and the frequency was 15 Hz with water-air cooling. When performing tooth extractions, the tooth alveolus was treated with an erbium laser in the hard tissue mode, and then the surrounding soft tissues of the adjacent gums and periodontal ligaments were treated with radiation from the erbium laser in the corresponding mode (Fig. 1B-D). This set was chosen according to the results of our previous experience of treatment of a patient with the same co-morbidity but in the case of periostitis [33]. Local hemostatic drugs were not used; preoperative drug preparation of patients was not performed. The control of hemostasis was carried out within 40 minutes after the intervention. In patients of the control group, surgical treatment was carried out according to the traditional method, without the use of laser technology.

On days 1 and 3 of the postoperative period, the severity of pain was evaluated using a digital rating scale (Numerical Pain Scale) (0–4 points: 0 indicates absence of pain, 1 lack of pain, 2-mild pain, 3-moderate pain, and 4-severe pain) and visual assessment of collateral edema (0–4 points: 0 indicates absence of edema, 1 lack of edema, 1 - mild edema, 2-moderate edema, 3-severe edema). We supposed the possibility of postoperative bleeding in points (0 indicates absence of bleeding, and 1 indicates bleeding and requiring treatment).

### 2.1. Statistics

We checked normality with the Shapiro–Wilcoxon test and used methods of non-parametric statistics because of the lack of normality of distributions. We compared the two groups according to each criterion with the help of Mann–Whitney test imagining that the null hypothesis μ_0_ was not equal to μ_1_ when μ_0_ “there were no differences between the groups”. We evaluated the differences in dynamics criteria, pain, and edema with the help of Kruskal–Wallis test and the probability of postoperative bleeding in both groups using the chi-square test. We analyzed the strength of correlation between different criteria and methods of treatment in both groups with the Pearson correlation coefficient in program R (designed by Ross Ihaka and Robert Gentleman, licensed in R Foundation, project GNU GPL, 2020, version 3.6.1) with installed packages *ggpubr* and *ggplot2*.

## 3. Results

The age of patients in both groups was similar and varied from 19 to 74 years in the 1^st^ group and from 19 to 81 years in the 2^nd^ group (U = 1020.5, p > 0.05).

All patients of the 1^st^ group had anxiety and fear not only of the upcoming treatment but also of the examination. Patients were not afraid of the manipulations, but of bleeding during the intervention and in the postoperative period. While the medical history, it was found that 11 (22.9%) patients of the first group noted prolonged bleeding, sometimes up to 2 days, after previous dental interventions (tooth extraction and professional oral hygiene). In two cases (4.2%), it was precisely such type of bleeding that was the first manifestation of a disease of the hematopoietic system two (4.2%) patients who underwent surgery were hospitalized with alveolar socket bleeding for emergency medical care.

We observed unsatisfactory oral hygiene in all patients with platelet hemostasis pathology since they were afraid of trauma to the gingival mucosa and subsequent bleeding during teeth brushing.

The characteristics of both groups are shown in Table 2.

The platelet count in patients with thrombocytopenia ranged from single to 140,000/μL (70 ± 27). In 16 patients (33.3%), an increase in bleeding time according to Duke was noted. Duke express test results did not always correlate with platelet count, which suggested that the risk of bleeding was often due to functional platelet disorders. Conducting express tests immediately before surgery allowed us to assess the degree of violation of platelet-vascular hemostasis at a given time.

Drug preparation before surgery was performed only in 7 (14.6%) patients with pathology of platelet hemostasis. In 3 (6.3%) of them, it consisted comprised administering intravenous drugs before surgical treatment because of severe thrombocytopenia against the background of ongoing chemotherapy and the urgency of the intervention. In 4 (8.3%) patients, drug preparation consisted of prescribing tablet forms of drugs (tranexamic acid) 3 days before surgical treatment and was due to a high risk of bleeding associated with a severe degree of hematopoietic system pathology, which is a manifestation of hemorrhagic syndrome.

During surgery, in all cases, in patients with platelet hemostasis disorders, increased bleeding from the vessels of the microvasculature of the soft tissues was defined, and the severity of bleeding was more often, but not always, correlated with the number of platelets in the peripheral blood.

The use of an erbium laser made it possible to achieve reliable hemostasis in all cases. The average value of hemostasis time when using a laser was 80.9 ± 35.9 seconds, the minimum and maximum values were 15 seconds and 175 seconds, respectively. With the traditional intervention technique, the average hemostasis time was 175 ± 67.5 seconds, with the minimum and maximum hemostasis times being 95 seconds and 300 seconds, respectively (p < 0.01).

Thus, when an erbium laser was used during the intervention, bleeding stopped in a shorter time than with the traditional method of intervention in patients without pathology of the hematopoietic system. No bleeding in the postoperative period was observed, apart from 1 case (2,3%) associated with violations of recommendations. Hemostasis was monitored at the clinic for 40 minutes (Table 3).

According to the results of the data obtained, during operations performed using an erbium laser, pain (Table 4) (H = 88.8, p < 0.001) and collateral edema (Table 5) (H = 74.8, p < 0.001) were both less pronounced on days 1 and 3 after surgery.

An important fact was that patients of the 1^st^ group did not take non-steroidal anti-inflammatory drugs due to the absence of a pronounced pain syndrome, which was especially important in patients with hemostatic disorders and in patients with erosive-ulcerative lesions of the gastrointestinal tract. It should be noted that during surgical treatment with an erbium laser, an increase in collateral edema from 1 to 3 operating days was not observed and an increase in edema was observed only in patients of the control group. We had not found the correlation between pain and edema in 2^nd^ group (r = 0.15, p > 0.05) with statistical significance and found connection between these criteria in the 1^st^ group (r = 0.54, p < 0.05). There was no correlation between time bleeding according to Duke and edema on the day 1 after operation in the 1^st^ group (r = −0.14, p > 0.05). The correlation between Morawitz time coagulation and edema on the day 1 after operation was rather weak (r = 0.29, p < 0.05). The correlation between the number of thrombocytes in the patients of the 1^st^ group and postoperative bleeding was not significant (r = 0.11, p > 0.05).

We also noted faster epithelization of wounds and sockets in this group of patients (on average, 2 days faster than in the 2^nd^ group), without signs of inflammation and without scar formation, despite the fact that in patients with diseases of the hematopoietic system, repair and regeneration potentials were reduced due to the concomitant pathology itself and the use of drugs prescribed for its treatment and correction of the condition.

## 4. Discussion

When providing surgical dental care to patients with platelet hemostasis disorders, an assessment of the risk of intra- and postoperative bleeding development is relevant. Several authors described various algorithms for preparing such patients for dental treatment - the use of thrombopoiesis stimulants and preparation using corticosteroid drugs [34–36]. It is recommended to perform simple tooth extraction with a platelet count of more than 30,000/μL, and more complex surgical interventions may be conducted with a platelet count of more than 50,000/μL [34,35]. However, these solutions require time and dynamic control of platelet count and their functional activity. At the same time, in oral surgery, we often encounter acute pain complaints and we need to perform surgical procedures without prolonged medical preparation.

Algorithms for assisting this group of patients and the results of studies conducted in this direction indicate the frequent need for surgical dental interventions in a hospital setting [34,35].

To optimize the care of patients with this pathology, we evaluated the possibility of using laser technologies on them. Dissecting the tissue, the laser beam simultaneously coagulates the vessels on the walls of the wound, contributing to hemostasis. In addition, postoperative edema is minimal, the intensity of intraoperative and postoperative pain is reduced, and patients do not experience psychoemotional stress [36–43].

In several studies, the opportunity of diodeportunity of diode and Nd: YAG laser was shown to stimulate periodontal regeneration of the tissues during the operation of tooth transplantation on the cell level because the increase in calcium deposit in osteoblasts and collagen in the fibroblasts leads to decrease in the time of wound healing [44].

There are no clinical studies on the use of erbium laser in the treatment of dental patients with the impaired hemostasis, but there are several studies in vivo; for example, Campos et al. (2018) compared the results of the use of high-power lasers and traditional scalpel in the rats after the administration of anticoagulants and created conditions of intensive bleeding during frenectomy of tongue that was completely solved with the laser radiation [30]. The same scientists pointed to the lack of evidence of basic information on the use of any lasers in patients with intra- and postoperative high risk of bleeding during oral surgery. Despite the possible priority of the laser energy for hemostasis, the complicity of tooth extraction in patients includes possible bleeding not only from soft tissues but also from the bottom and alveolar walls, which require other working modes of laser.

The use of erbium can significantly reduce the risk of late bleeding in patients with impaired platelet hemostasis, making it possible to carry out surgical manipulation in an outpatient dental setting without prolonged medical preparation and provide stimulation of alveolar epithelization and reduce the number of pathogenic microorganisms. In some cases, the use of an erbium laser successfully solves the problem of providing emergency surgical dental care even in patients with resistant forms of the disease and the manifestation of hemorrhagic syndrome [45–49].

## Supplementary Information











## Figures and Tables

**Table 1:** The inclusion and exclusion criteria for patients.

Criteria	Inclusion	Exclusion
Age, years	>18	<18
Thrombocytes/μL	Approved thrombocytopenia (<100)	Reference means
Medical treatment of thrombocytopenia	Permanent	Periodical
Indications for oral surgery	Present	-
Other comorbidities, besides thrombocytopenia	Absent	Present
Compliance	Good	Bad

**Table 2:** Characteristics of hemostasis and age of patients in the 1^st^ and 2^nd^ groups.

Characteristic	1^st^ group Mean ± SD (Median, Min–Max)	2^nd^ group Mean ± SD (Median, Min–Max)	p
Age, years	44 ± 15.4 (42, 19–74)	47.6 ± 15.3 (49, 19–81)	>0.05
Trombocytes/μL	70 ± 27 (65, 1–140)	304 ± 114 (315, 200–435)	<0.05
Morawitz coagulation time, min	7,6 ± 0.67 (6.6, 4.1–17)	-	-
Duke bleeding time, min	5,38 ± 2.6 (5.35, 4.1–7.1)	-	-

* SD-standard deviation, p-p-value.

**Table 3:** Characteristics of postoperative hemostasis in the 1^st^ and 2^nd^ groups.

Characteristic	1^st^ group Mean ± SD (Median, Min–Max)	2^nd^ group Mean ± SD (Median, Min–Max)	P
Hemostasis time after operation, sec	80.9 ± 35.9 (78, 15–175)	175 ± 67.5 (170, 95–300)	<0.01
Postoperative bleeding, points	0.02 ± 0.14 (0, 0–1)	0 (0, 0)	<0.05

**Table 4:** The distribution of study patients in the 1^st^ and the 2^nd^ groups according to the severity of pain in the postoperative period.

The severity of pain	Study group 1^st^ N (%)		Study group 2^nd^ N (%)	
		
	Day 1	Day 3	Day 1	Day 3
Absence of pain	3 (6.3%)	34 (70.8%)	-	8 (16.7%)
Lack of pain	24 (50%)	13 (27.1%)	9 (18.8%)	24 (50%)
Mild pain	13 (27.1%)	1 (2.1%)	14 (28.6%)	14 (28.6%)
Moderate pain	5 (10,3%)	—	17 (35.9%)	2 (4.7%)
Severe pain	3 (6.3%)	—	8 (16.7%)	—
p-value	<.001			

**Table 5:** The distribution of study patients in the 1^st^ and the 2^nd^ groups according to the severity of edema in the postoperative period.

The severity of collateral edema	Study group 1^st^ N (%)		Study group 2^nd^ N (%)	
		
	Day 1	Day 3	Day 1	Day 3
Absence of edema	29 (60.4%)	40 (83.3%)	4 (8.3%)	4 (8.3%)
Lack of edema	13 (27.1%)	8 (16.7%)	29 (60.4%)	20 (41.2%)
Mild edema	4 (8.3%)	—	12 (25%)	10 (20.6%)
Moderate edema	1 (2.1%)	—	3 (6.3%)	14 (29.9%)
Severe edema	1 (2.1%)	—	—	—
p-value	<.001			
