# Supplementary material for: Laser-assisted tooth extraction in patients with impaired hemostasis
Source: Biomedicine (Taipei). 2021 Jun 1;11(2):47–54. doi: 10.37796/2211-8039.1072 (PMC8824248; doi:10.37796/2211-8039.1072)
Supplement: Supplementary file 1 [file bmed-11-02-047-s001.pdf]

BioMedicine

AUTHORSHIP STATEMENT

Article LASER-ASSISTED TOOTH EXTRACTION IN PATIENTS WITH IMPARED HEMOSTASIS

All persons who meet authorship criteria are listed as authors, and all authors certify that theyhave participated sufficiently in the work to take public responsibility for the content, including participation in the concept, design, analysis, writing, or revision of the manuscript. Furthermore, each author certifies that this material or similar material has not been and will not be submitted to or published in any other publication before its appearance in BioMedicine.

Authorship contributions

Please indicate the specific contributions made by each author (list the authors' initials followed by their surnames, e.g., Y.L. Chang). The name of each author must appear at least once in each of the three categories below.

Category 1

Conception and design of study: Larionova E.V., Diachkova E.Yu., ____, ____; ____;

acquisition of data: Larionova E.V., Morozova E.A., Davtyan A.A., ____; ____;

analysis and/or interpretation of data: Diachkova E.Yu., Larionova E.V., Tarasenko S.V., ____. ____;

Category 2

Drafting the manuscript: Diachkova E.Yu., Moozova E.A., ____, ____;

revising the manuscript critically for important intellectual content:

Tarasenko S.V., ____, ____, ____.

Category 3

Approval of the version of the manuscript to be published (the names of all authors must be listed):

Tarasenko Svetlana, Morozova Elena, Larionova Elena, Diachkova Ekaterina,

Davtyan Albert, ____, ____, ____,

Acknowledgments

All persons who have made substantial contributions to the work reported in the manuscript (e.g., technical help, writing and editing assistance, general support), but who do not meet the criteria for authorship, are named in the Acknowledgments and have given us their written permission to be named. If we have not included an Acknowledgments, then that indicates that we have not received substantial contributions from non-authors.

This statement is signed by all the authors (a photocopy of this form may be used if there are more than 10 authors):

Author's name (typed)	Author's signature	Date
Ekaterina Diachkova	Подписано цифровой подписью: Ekaterina Diachkova Дата: 2020.04.20 15:22:32 +03'00'	04/20/2020
Svetlana Tarasenko	[signature]	04/20/2020

Elena Larionova		04/20/20
Elena Morozova		04/20/20
Albert Davtyan		04/20/20

Corresponding author declaration

I [Diachkova E.Y.] , the corresponding author of this manuscript, certify that the contributors' and conflicts of interest statements included in this paper are correct and have been approved by all co-authors
