# Supplementary material for: Laser-assisted tooth extraction in patients with impaired hemostasis
Source: Biomedicine (Taipei). 2021 Jun 1;11(2):47–54. doi: 10.37796/2211-8039.1072 (PMC8824248; doi:10.37796/2211-8039.1072)
Supplement: Supplementary file 3 [file bmed-11-02-047-s004.pdf]

федеральное государственное автономное образовательное учреждение высшего образования
Первый Московский государственный медицинский университет имени И.М. Сеченова
Министерство здравоохранения Российской Федерации
(Сеченовский Университет)

Сеченовский Университет

ЛОКАЛЬНЫЙ ЭТИЧЕСКИЙ КОМИТЕТ

119991, г. Москва, ул. Трубецкая, д. 8 тел.: 8(495)622-97-06, факс: 8(495)622-97-56, iec@1msmu.ru; iec@sechenov.ru

**Выписка из протокола № 14-19
заседания
Локального этического Комитета
от 13.11.2019**

<u>Присутствовали:</u>
Председатель Комитета - Николенко В.Н.
Заместитель председателя Комитета – Реброва Е.Л.
Члены Комитета: Арсланян К.С., Ермолаева И.И., Бердникова Н.Г., Борисова Н.И., Дубограй Е.В., Смолярчук Е.А., Субботина О.А.

Кворум есть, заседание считается правомочным.

Слушали: рассмотрение исследования в рамках диссертационной работы «Применение лазерного ассистирования при удалении зубов у пациентов, принимающих антиагрегантные препараты» (исполнитель – Давтян Альберт Артемович).

Постановили: одобрить исследование в рамках диссертационной работы «Применение лазерного ассистирования при удалении зубов у пациентов, принимающих антиагрегантные препараты» (исполнитель – Давтян Альберт Артемович).

Выписка верна.

Ответственный секретарь И.И. Ермолаева

17.01.2019

Локальный КОМИТЕТ ПО ЭТИКЕ
ФГАОУ ВО Первый МГМУ им. И.М. Сеченова
Минздрава России (Сеченовский Университет)
Т. 8(495)622-97-06

<u>*Перевод с русского языка на английский язык*</u>
<u>*Translation from Russian into English*</u>

Federal State Autonomous Educational Institution of Higher Education
I.M. Sechenov First Moscow State Medical University
of the Ministry of Health of the Russian Federation
(Sechenov University)

/Logo: Sechenov University of Life Sciences/

INSTITUTIONAL ETHICS COMMITTEE

119991, city of Moscow, ul. Trubetskaya, d. 8 Phone: 8(495)622-97-06, fax: 8(495)622-97-56, iec@1msmu.ru, iec@sechenov.ru

Extract from Minutes No. 14-19
of the Meeting of
the Institutional Ethics Committee
dated 13 November 2019

<u>Present:</u>
V.N. Nikolenko, Chairman of the Committee
E.L. Rebrova, Deputy Chairman of the Committee
Members of the Committee: K.S. Arslanyan, I.I. Yermolayeva, N.G. Berdnikova, N.I. Borisova, Ye.V. Dubograi, E.A. Smolyarchuk, O.A. Subbotina

A quorum is present, so the meeting shall be deemed duly constituted.

Heard: approval of the study as part of the dissertation "Use of laser-assisted tooth extraction in patients taking antiplatelet drugs" (prepared by Albert Artemovich Davtyan).

Resolved: to approve the study as part of the dissertation "Use of laser-assisted tooth extraction in patients taking antiplatelet drugs" (prepared by Albert Artemovich Davtyan).

True extract.

Executive Secretary */Signature/* I.I. Yermolayeva

17 January 2019

/Stamp: Institutional Ethics Committee
Federal State Autonomous Educational Institution of Higher Education
I.M. Sechenov First Moscow State Medical University
of the Ministry of Health of Russia (Sechenov University)
Phone: 8(495)622-97-06/

Перевод настоящего документа с русского языка на английский язык выполнен мной, переводчиком Левченко Юлией Васильевной. Верность перевода подтверждаю.

_______________ *(Левченко Ю.В.)*

Бюро переводов
ООО «Монблан»
Филиал Таганская
Москва, Таганская площадь, д. 86/1, стр. 1
Тел. +7 495 120 20 96

Сертификат соответствия
Международной системе
менеджмента качества
ISO 9001:2015

The document has been translated from Russian into English by me, Yulia Vasilievna Levchenko. I hereby certify the correctness of the translation.

_______________ *(Y.V. Levchenko)*

Translation Agency
Monblan LLC
Taganskaya Branch
Moscow, 86/1 Taganskaya ploshchad, bldg 1
Tel. +7 495 120 20 96

Certificate of compliance
with International
Quality Management System
ISO 9001:2015

*/Official seal: Moscow * MONBLAN, Limited Liability Company*
Primary State Registration Number (OGRN) 1097746313455
For documents 4/
