# Supplementary material for: Laser-assisted tooth extraction in patients with impaired hemostasis
Source: Biomedicine (Taipei). 2021 Jun 1;11(2):47–54. doi: 10.37796/2211-8039.1072 (PMC8824248; doi:10.37796/2211-8039.1072)
Supplement: Supplementary file 4 [file bmed-11-02-047-s005.pdf]

<u>**Министерство здравоохранения и социального развития.**</u>

ГОСУДАРСТВЕННОЕ ОБРАЗОВАТЕЛЬНОЕ УЧРЕЖДЕНИЕ ВЫСШЕГО ПРОФЕССИОНАЛЬНОГО ОБРАЗОВАНИЯ МОСКОВСКИЙ ГОСУДАРСТВЕННЫЙ МЕДИКО-СТОМАТОЛОГИЧЕСКИЙ УНИВЕРСИТЕТ (ГОУ ВПО МГМСУ)

КОМИТЕТ ПО ЭТИКЕ

Выписка из протокола заседания №6 от 22 февраля 2011 г.

<u>**Присутствовали.**</u>

Председатель Комитета по этике: д.м.н., профессор Маев И.В.
Зам. председателя Комитета по этике: д.м.н., профессор Майчук Е.Ю.
Члены Комитета по этике: Карамышева Е.И. (ответственный секретарь), Ющук Н.Д., Малый А.Ю., Изюмов Е.Г., Кузьмин В.Н., Гацура С.В., Лукьянова И.Ф., Вольская Е.А., Теблоев К.И.

<u>**Слушали:**</u> рассмотрение вопроса об одобрении клинического исследования в рамках диссертационной работы на тему: «Хирургическое стоматологическое лечение пациентов с нарушениями тромбоцитарного звена гемостаза с помощью лазеров».
Исполнитель: Макарова Е.В.

<u>**Постановили:**</u> одобрить клиническое исследование в рамках диссертационной работы на тему: «Хирургическое стоматологическое лечение пациентов с нарушениями тромбоцитарного звена гемостаза с помощью лазеров».
Исполнитель: Макарова Е.В.

Голосовали:
за - 11, против - нет; воздержались - нет.

Решение принято единогласно.

Председатель
Комитета по этике
д.м.н., профессор И.В. Маев

Ответственный секретарь
Комитета по этике
д.м.н., профессор Е.И. Карамышева

Ministry of Health and Social Development

STATE EDUCATIONAL INSTITUTION OF HIGHER PROFESSIONAL EDUCATION
MOSCOW STATE UNIVERSITY OF MEDICINE AND DENTISTRY (SEI HPE MSUMD)

ETHICS COMMITTEE

Extract from Minutes of the Meeting No. 6 dd. 22 February 2011

Present:

Chairman of the Ethics Committee: Dr. habil., Prof. I.V. Mayev

Deputy Chairman of the Ethics Committee: Dr. habil., Prof. E.Yu. Maichuk

Members of the Ethics Committee: E.I. Karamysheva (Executive Secretary), N.D. Yushchuk, A.Yu. Maly, E.G. Izyumov, V.N. Kuzmin, S.V. Gatsura, I.F. Lukyanova, E.A. Volskaya, K.I. Tebloev

Agenda: Consideration of the question on approval of a clinical trial as part of a dissertation on: "Laser Surgical Dental Treatment of Patients with Platelet Hemostasis Disorders".

Investigator: E.V. Makarova

Resolved: To approve a clinical trial as part of a dissertation on: "Laser Surgical Dental Treatment of Patients with Platelet Hemostasis Disorders".

Investigator: E.V. Makarova

Voted:

"For" – 11, "Against" – no, "Abstain" – no.

The resolution was adopted unanimously.

Chairman
of the Ethics Committee
Dr. Habil., Prof. *Signature/* I.V. Mayev

Executive Secretary
of the Ethics Committee
Dr. Habil., Prof. */Signature/* E.I. Karamysheva

Перевод настоящего документа с русского языка на английский язык выполнен мной, переводчиком Левченко Юлией Васильевной. Верность перевода подтверждаю.

_______________ *(Левченко Ю.В.)*

Бюро переводов
ООО «Монблан»
Филиал Таганская
Москва, Таганская площадь, д. 86/1, стр. 1
Тел. +7 495 120 20 96

Сертификат соответствия
Международной системе
менеджмента качества
ISO 9001:2015

ОБЩЕСТВО С ОГРАНИЧЕННОЙ ОТВЕТСТВЕННОСТЬЮ ОГРН 1097746313455 МОСКВА
«МОНБЛАН»
для документов
4

The document has been translated from Russian into English by me, Yulia Vasilievna Levchenko. I hereby certify the correctness of the translation.

_______________ *(Y.V. Levchenko)*

Translation Agency
Monblan LLC
Taganskaya Branch
Moscow, 86/1 Taganskaya ploshchad, bldg 1
Tel. +7 495 120 20 96

Certificate of compliance
with International
Quality Management System
ISO 9001:2015

*/Official seal: Moscow * MONBLAN, Limited Liability Company*
Primary State Registration Number (OGRN) 1097746313455
For documents 4/
